# Persistent Histamine Excitation of Glutamatergic Preoptic Neurons

**DOI:** 10.1371/journal.pone.0047700

**Published:** 2012-10-17

**Authors:** Iustin V. Tabarean

**Affiliations:** The Department of Molecular and Integrative Neurosciences, The Scripps Research Institute, La Jolla, California, United States of America; University of Pecs Medical School, Hungary

## Abstract

Thermoregulatory neurons of the median preoptic nucleus (MnPO) represent a target at which histamine modulates body temperature. The mechanism by which histamine excites a population of MnPO neurons is not known. In this study it was found that histamine activated a cationic inward current and increased the intracellular Ca^2+^ concentration, actions that had a transient component as well as a sustained one that lasted for tens of minutes after removal of the agonist. The sustained component was blocked by TRPC channel blockers. Single-cell reverse transcription-PCR analysis revealed expression of TRPC1, TRPC5 and TRPC7 subunits in neurons excited by histamine. These studies also established the presence of transcripts for the glutamatergic marker Vglut2 and for the H1 histamine receptor in neurons excited by histamine. Intracellular application of antibodies directed against cytoplasmic sites of the TRPC1 or TRPC5 channel subunits decreased the histamine-induced inward current. The persistent inward current and elevation in intracellular Ca^2+^ concentration could be reversed by activating the PKA pathway. This data reveal a novel mechanism by which histamine induces persistent excitation and sustained intracellular Ca^2+^ elevation in glutamatergic MnPO neurons.

## Introduction

Histamine controls arousal, attention, feeding and thermoregulation (reviewed in [1]). Histaminergic fibers originating from the tuberomammilary nucleus are dense in the median preoptic nucleus [2], one of the sites containing thermoregulatory neurons (reviewed in [3]). Histamine injected in the median preoptic nucleus (MnPO) induces long lasting hyperthermia (at least 6 hours) in several mammalian species studied [4], [5], [6]. Yet, in the brain histamine is quickly degraded by the activity of the histamine-N-methyltransferase. In the hypothalamus the neurotransmitter has a half-life in the order of minutes [7], [8] suggesting that its long lasting effects may reflect sustained changes in neuronal activity.

While the role of tonic inhibition of thermoeffector controlling neurons by GABAergic MnPO neurons is well documented (reviewed in [9]) more recently it has been revealed that thermoregulation can be directly modulated by glutamatergic MnPO neurons [10], [11] and by glutamatergic hypocretin-expressing neurons of the lateral hypothalamus [12]. In a previous study we have identified two distinct mechanisms by which histamine modulates the activity of MnPO neurons and core body temperature: inhibition of GABAergic neurons expressing H3 receptors and excitation of non-GABAergic neurons expressing H1 receptors [10]. We have further determined that histamine decreases the firing rate of GABAergic neurons by augmenting an A-type current conducted by Kv4.2-containing channels [6]. The ionic mechanism involved in the depolarization induced by H1 receptor activation is not known. In other preparations, activation of H1 receptors results in depolarization and increased firing rate by either the activation of a cationic current [13], [14], [15] or by a decrease in a K leak conductance [16], [17], [18], [19]. TRPC channels conduct cationic currents and are commonly involved in signaling pathways downstream of Gq coupled receptors, such as the H1 receptor. The present study investigates the ionic mechanisms of histamine depolarization of identified glutamatergic MnPO neurons and the role of TRPC channels in these actions.

## Materials and Methods

### Ethics Statement

All animal work was conducted in accordance with the Institutional Animal Care and Use Committee of the Scripps Research Institute (approval ID #08-0129). The standards are set forth by American Association for the Accreditation of Laboratory Animal Care (AAALAC) standards and the regulations set forth in the Animal Welfare Act.

**Table 1:** Primers.

Primer	External sequence	Amplicon size	Internal sequence	Ampliconsize
**TRPC1**	F: 5′ tctggaatgttcctccttg 3′;R: 5′gctcgagcaaacttccattc 3′	Bp:375	F: 5′ accttccactcgttcattgg 3′R: 5′ tcagctggaaggtcttg 3′	Bp:208
**TRPC2**	F:5′ gccagcaagttctgtcttcc3′R:5′atgccaagtacagggacagg3′	Bp:429	F: 5′ tgttgccttcctcatcttcc 3′R:5′ accagagactctcccagcaa 3′	Bp:165
**TRPC3**	F:5′ gctggccaacatagagaagg3′R:5′ acgtgaactgggtggtcttc3′	Bp:556	F:5′ gcacaaagcgtcactgagtc 3′R: 5′ccggaaaggttctcatacca 3′	Bp:117
**TRPC4**	F: 5′: gttgggagctgcaagaactc 3′R:5′ gacctgtcgatgtgctgaga 3′	Bp:499	F: 5′gacacggagttccagagagc3′R:5′ acagtccaagtgggcttttg 3′	Bp:299
**TRPC5**	F: 5′: cgctctttgaaacccttcag 3′R:5′ gacaggcctttttcttgcag 3′	Bp: 729	F: 5′: ggaggcacaacttgagaagc3′R:5′ tggagaggcttcttgga 3′	Bp:243
**TRPC6**	F: 5′: tccaaactgtcagcaacagc 3′R:5′ cagcattccaaagtcaagca3′	Bp: 463	F: 5′: tactggtgtgctccttgcag3′R:5′ cagctgccttgcattatctg 3′	Bp:180
**TRPC7**	F:5′ ccctgggaagaactgtgaaa 3′R:5′ cctggtagcgagtcttcctg 3′	Bp: 567	F: 5′: ccagcgtttacacagttga 3′R:5′ gctcgagcaaatttccactc 3′	Bp:245

### Slice Preparation

Coronal tissue slices containing the median preoptic nucleus (MnPO) were prepared from C57/Bl6 mice (28–42 days old) housed in standard conditions. An animal was anesthetized using isoflurane and sacrificed by decapitation, according to procedures approved by the Animal Welfare Committee of the Scripps Research Institute. Brain slices were prepared as previously described [10]. The slices used in our recordings corresponded to the sections located from 0.5 mm to 0.26 mm rostral to Bregma in the mouse brain atlas [20]. The slices were prepared at 9–11 am local time during the “subjective light period” and recordings were carried out up to the end of this period i.e. 8 pm local time.

**Figure 1:**
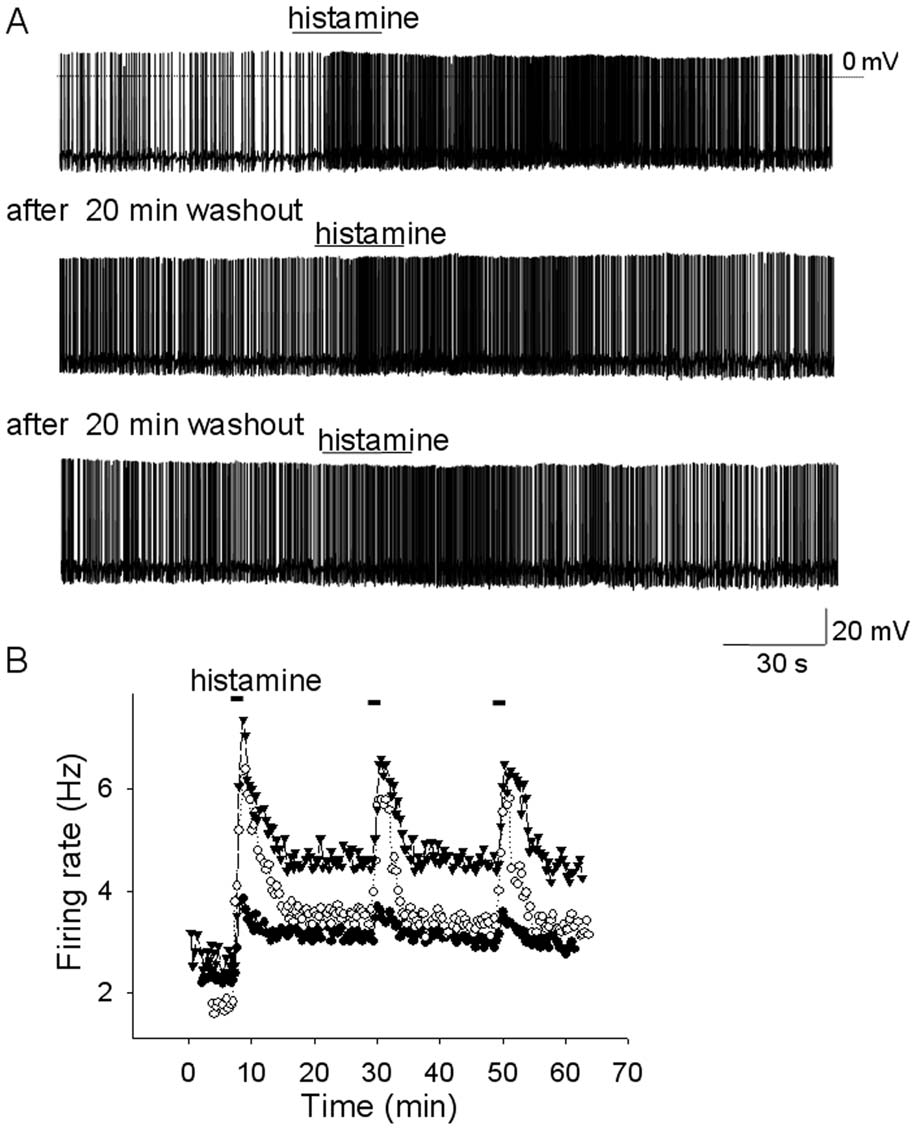
Histamine induces a persistent increase of the firing rate of MnPO neurons. A. Spontaneous firing activity recorded before and during local application of histamine (50 µM). Histamine induced a depolarization (∼3 mV) and increased the firing rate from 1.7 Hz to 3.6 Hz. Histamine (50 µM) was reapplied twice following a ∼22 min washout period after each application (middle and lower traces). Note that after the first histamine incubation the firing rate recovered only partially and remained elevated throughout the experiment. B. Average firing rate (for every 20 s) recorded before, during and after three incubations with histamine (50 µM), separated by ∼22 min washout periods. Data are from three MnPO neurons recorded in slices. The white circles (○) correspond to the experiment presented in A.

### Identification of Glutamatergic MnPO Neurons

Glutamatergic neurons represent a large proportion of MnPO neurons and are characterized by firing rates of 1–5 Hz, lower than those of GABAergic neurons [10]. This criterion was used for preliminary identification of glutamatergic neurons. Single cell RT/PCR analysis in a large number of recorded neurons (see below) has been carried out and it was confirmed that most (81%) slow firing neurons were Vglut2 positive. For these experiments the cytoplasm of the recorded neurons was aspirated at the end of the recording and stored at −80°C. Within 5 days after being harvested the cytoplasm samples from 6–10 neurons were individually and in parallel analyzed by RT/PCR.

**Figure 2:**
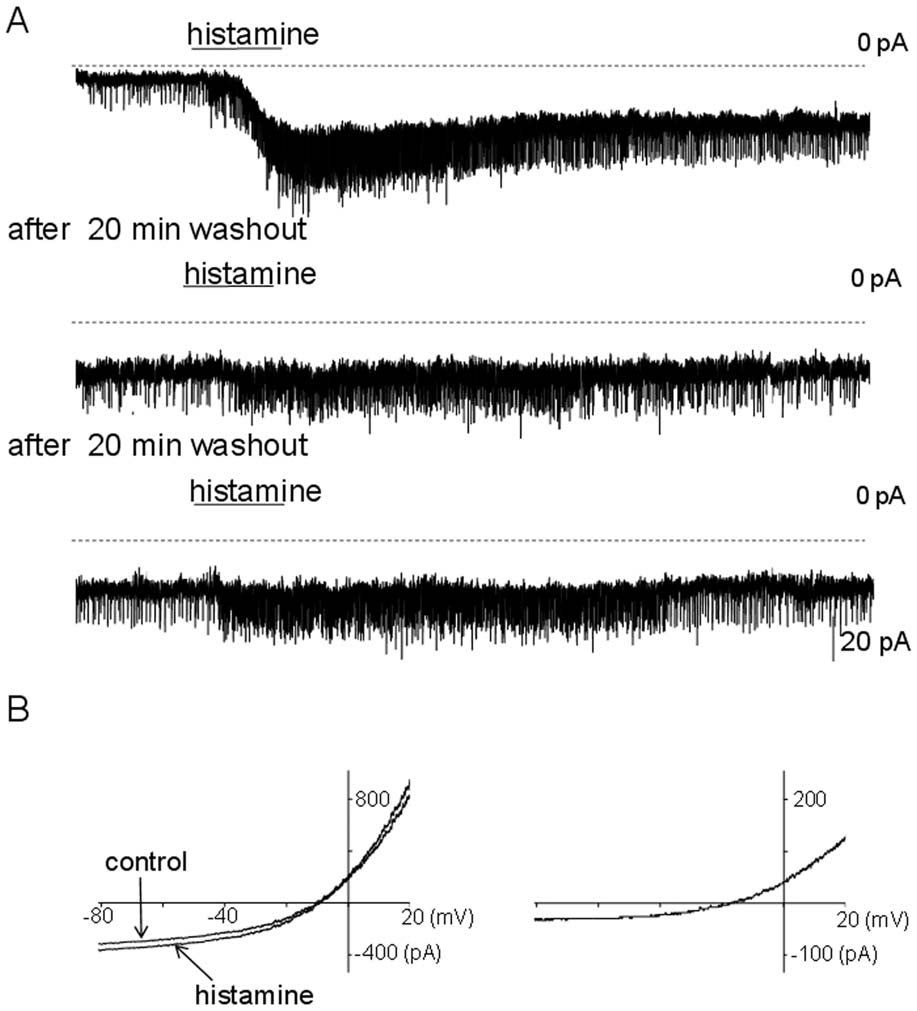
Histamine activates a persistent inward current. A. Application of histamine (50 µM) results in an inward current of ∼35 pA and an increase in the frequency of sEPSCs from 0.9 Hz to 5.3 Hz in a MnPO neuron recorded in a slice. The neuron was voltage-clamped at −50 mV. The histamine incubation (50 µM) was repeated twice following a ∼22 min washout period each time (middle and lower traces). Note that after the first histamine incubation the inward current and the sEPSC frequency recovered only partially and remained elevated throughout the experiment. B. I–V relationships between −70 and 30 mV in control and during histamine incubation (50 µM) (left) and their difference representing the histamine-evoked inward current (right). TTX (1 µM) was present in the extracellular solution. The duration of the ramp was 1s. the recording was from an acutely dissociated MnPO neuron.

### Dissociated Preoptic Neurons from Slices

The MnPO was punched out of a brain slice and incubated in Hibernate A (Invitrogen, Temecula, CA) and papain (Worthington, Lakewood, NJ) (1 mg/ml) for 10 min at room temperature. After washing out the enzyme with Hibernate –A the cells were dissociated by gentle trituration with a fire-polished Pasteur pipette. The cell suspension was pelleted (1000 g for 2 min) and resuspended in Neurobasal medium and then plated on poly-D-lysine/laminin coated coverslips (Biocoat, BD Biosciences, Bedford, MA). Cells were allowed to attach to the coverslips for 3–5 hours before recording.

**Figure 3:**
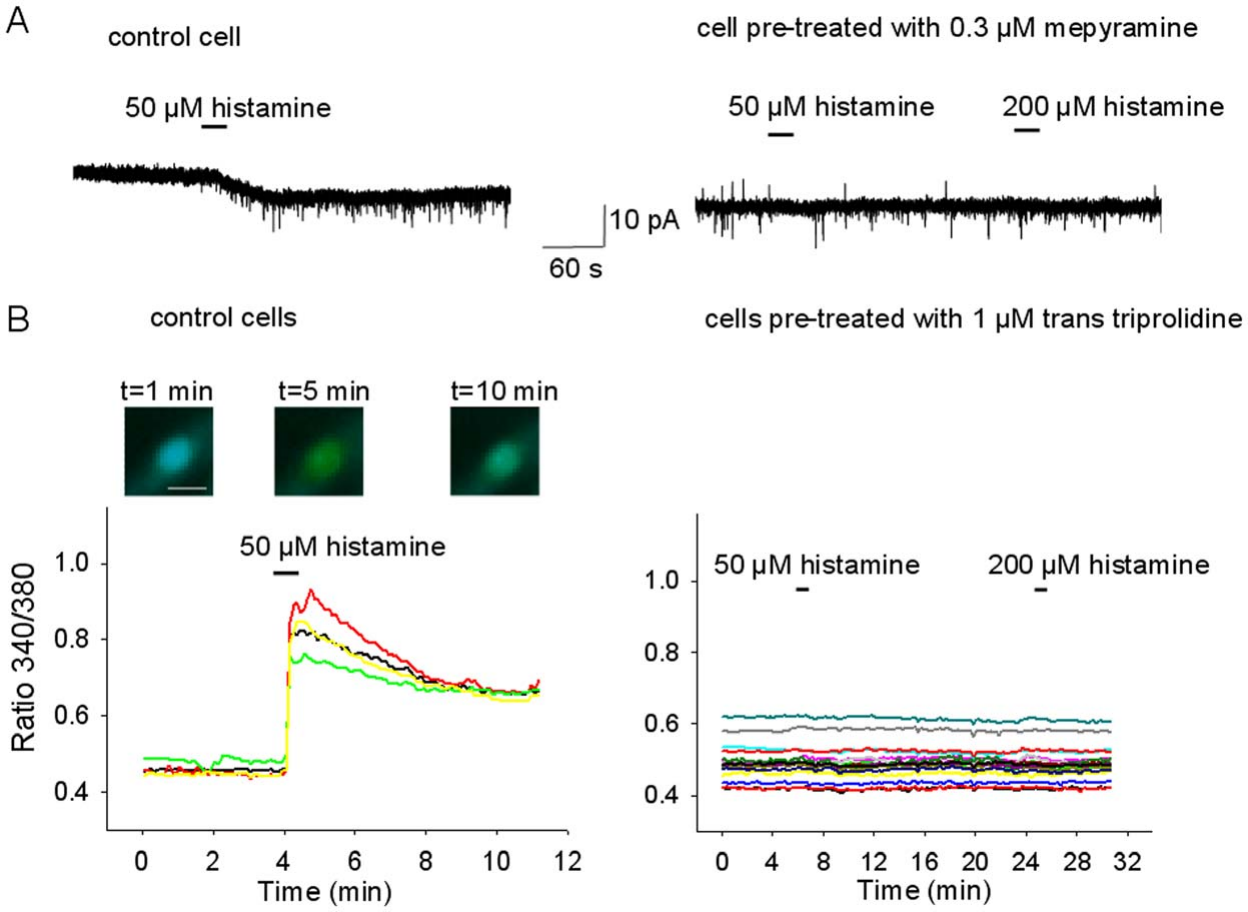
Histamine effects are abolished by pre-incubation with mepyramine or trans triprolidine. A. Representative recording of a histamine (50 µM) responses in a MnPO neuron in control conditions (left) and after pre-incubation with the H1R selective antagonist mepyramine (0.30 µM) for 5 min (right). B. Representative recordings of [Ca]_i_ responses to histamine (50 µM) from 4 acutely dissociated MnPO neurons in control conditions and after pre-incubation with the H1R antagonist trans triprolidine (1 µM) for 5 min (right, n = 16 neurons). Note that the antagonist abolishes the effects of histamine on [Ca]_i_. The insets above the graph are representative images of the Fura-2 ratio taken at the indicated time points from a MnPO neuron (red trace in the graph). Increasing levels of the intracellular [Ca]_i_, i.e. of the 340/380 ratio, are indicated by a change in color (blue-green-yellow). The lowest levels of [Ca^2+^]_i_ are indicated in blue. The scale bar represents 20 µm.

### Whole-cell Patch Clamp Recording

The artificial cerebrospinal fluid (aCSF) contained (in mM): 130 NaCI, 3.5 KCI, 1.25 NaH_2_PO_4_, 24 NaHCO_3_, 2 CaCI_2_, 1 MgSO_4_, 10 glucose osmolarity of 300–305 mOsm, equilibrated with 95% O_2_ and 5% CO_2_ (pH 7.4). In some experiments the aCSF was supplemented with TTX (1 µM). A K^+^ pipette solution containing (in mM) 130 K-gluconate, 5 KCI, 10 HEPES, 2 MgCI_2_, 0.5 EGTA, 2 ATP, 1 GTP (pH7.3) was used in most experiments. In some experiments a pipette solution in which EGTA was replaced with BAPTA (10 mM) was used. The electrode resistance after back-filling was 2–4 MΩ. All voltages were corrected for the liquid junction potential (−13 mV). The recording chamber was constantly perfused with aCSF (2–3 mL/min). The treatments were applied locally using a perfusion pencil system (tip diameter 100 µm, Automate Scientific) driven by gravity. Using this perfusion system the time interval required for the exchange of the solution bathing the recorded cell (measured using a high K solution) was less than 15 s. The temperature of the external solution was controlled with a TC-344B temperature controller and an inline heater (Warner Instruments, Hamden CT) and was maintained at 36–37°C. Mepyramine, *trans* triprolidine, KT5720, YM58483 and SKF 96265 were from Tocris (Ellisville, MO) while the other substances were purchased from Sigma. The TRPC1 (rabbit anti-human cat# ACC-010) TRPC3 (rabbit anti-mouse cat# ACC-016) and TRPC5 (rabbit anti-human cat# ACC-020) antibodies were purchased from Alomone Labs (Jerusalem, Israel). The specificity of these antibodies and their use as blocking antibodies has been discussed in other studies [21], [22], [23], [24], [25], [26].

**Figure 4:**
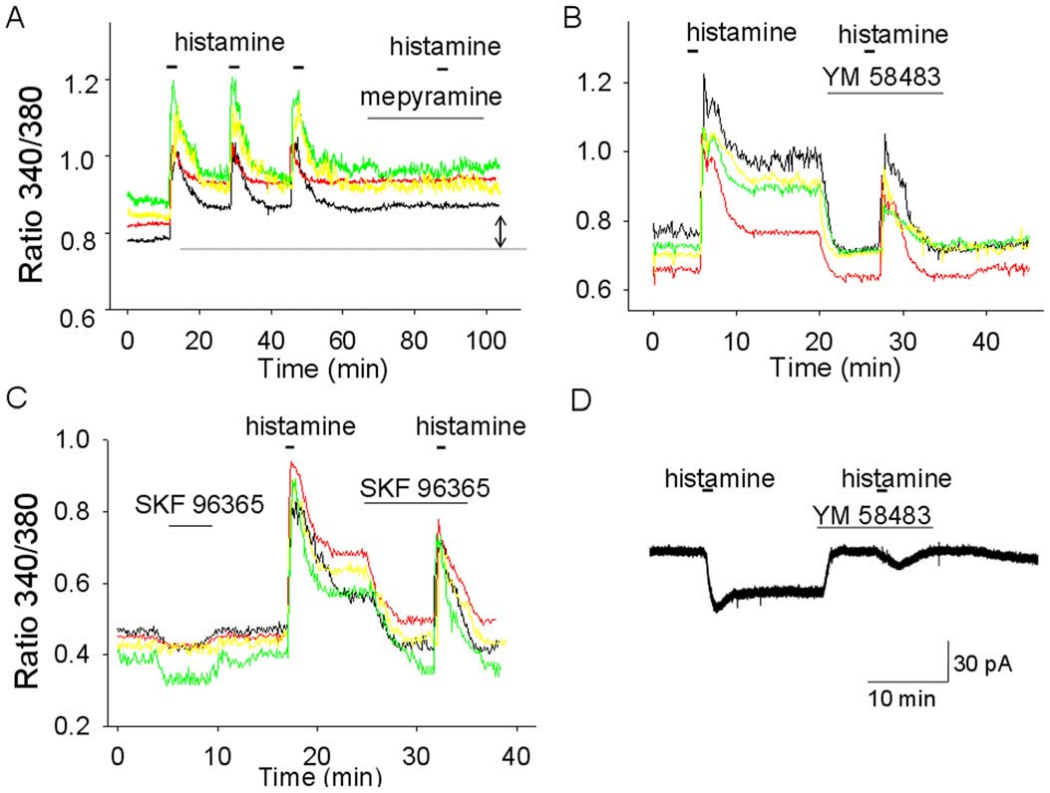
Histamine activates both transient and persistent [Ca]_i_ responses. A. [Ca]_i_ responses to histamine (50 µM) from 4 acutely dissociated MnPO neurons. The histamine treatment was then repeated twice following periods of washout of ∼20 min each. Note that after the first histamine incubation [Ca]_i_ recovered at an elevated level relative to the control (see double arrow). Incubation with the H1R selective antagonist mepyramine (0.50 µM) did not decrease the [Ca]_i_ plateau but abolished the transient increase activated by histamine. B. The TRPC channel blocker SKF96365 (100 µM) decreases the histamine activated [Ca]_i_ plateau but does not affect the transient increase activated by the neurotransmitter. Note that the blocker has little influence on basal [Ca]_I._ Data from 4 cells loaded with fura-2AM (black, red, green and yellow traces). C. The TRPC channel blocker YM 58483 (100 µM) decreases the histamine activated [Ca]_i_ plateau but does not affect the transient increase activated by the neurotransmitter. Data from 3 cells loaded with fura-2AM (red, green and yellow traces) and once cell, from a different experiment, loaded with fura-2 through the patch pipette (black trace). D. Simultaneous voltage-clamp and [Ca]_i_ recording from a MnPO neuron and the responses to the same treatment protocol as in B. YM 58483 (100 µM) blocks the persistent inward current activated by histamine does not affect the transient inward current activated by the neurotransmitter. The [Ca]_i_ response of the neuron is presented in B (black trace). A–D. TTX (1 µM) was added to all extracellular solutions.

### Data Acquisition and Analysis

Data was acquired with a MultiClamp 700B amplifier (Molecular Devices) digitized using a Digidata 1320A interface and the Pclamp9.2 software package. The sampling rate for the continuous recordings of spontaneous activity was 10 kHz. After establishing whole-cell configuration (or cell-attached) the spontaneous activity of the neuron was recorded for 2–4 min to determine its control behavior at (36–37°C). The spontaneous firing activity of the neurons was recorded in whole-cell mode (current clamp, I = 0). In voltage-clamp experiments the holding potential was −50 mV. Synaptic potentials were detected automatically with Mini Analysis (Synaptosoft). The frequency, amplitudes, and kinetics of synaptic events were analyzed using MiniAnalysis software. The statistical significance of differences between cumulative distributions was estimated with the Kolmogorov-Smirnov (K–S) parametric test. The values reported are presented as mean ± standard deviation (S.D.). Statistical significance of the results pooled from two groups of cells was assessed with *t*-tests using Prism4 software (Graphpad). One way analysis of variance (ANOVA) with Tukey-Kramer post hoc test (*P*<0.05) was used for comparison of multiple groups.

**Figure 5:**
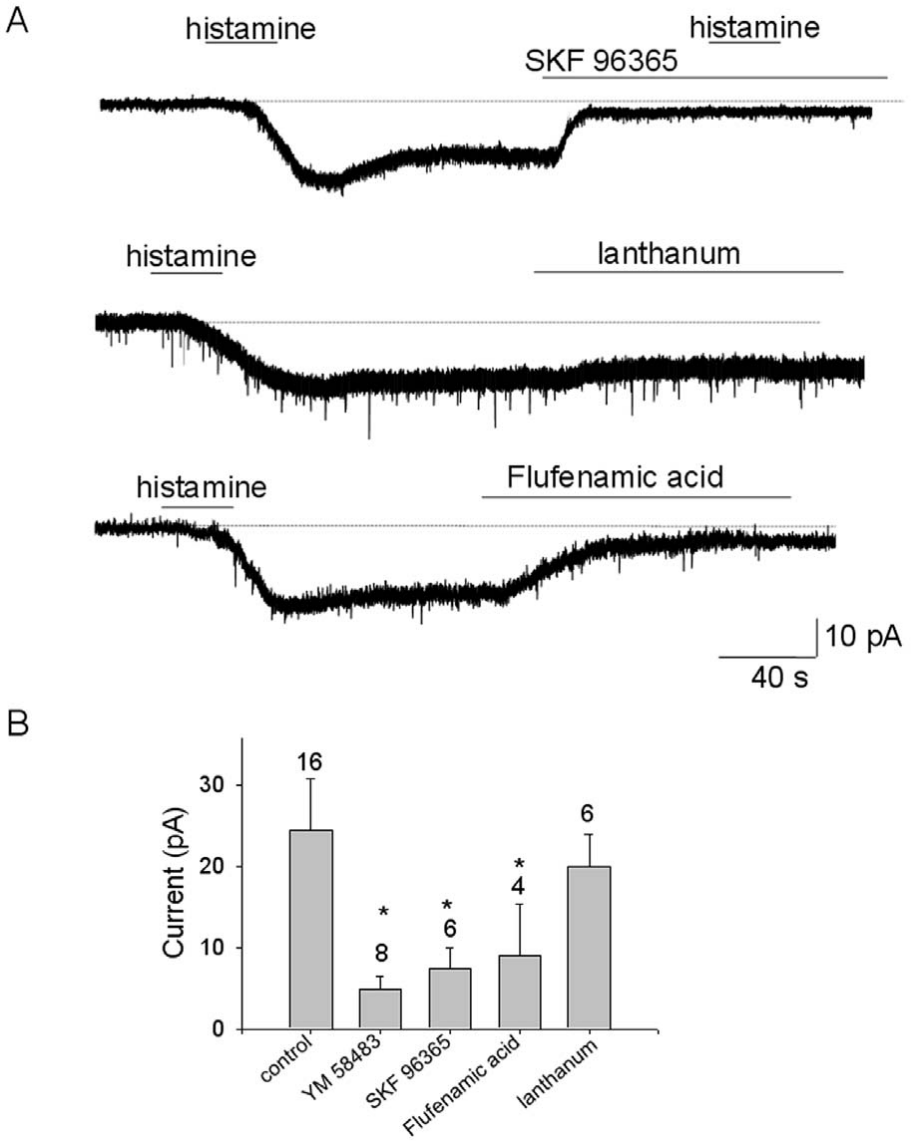
Pharmacology of the persistent inward current activated by histamine. A. Effects of SKF96365 (100 µM) (upper trace), lanthanum (100 µM) (middle trace) and flufenamic acid (100 µM) (lower trace) on the persistent inward current activated by histamine (50 µM). TTX (1 µM) was present in all extracellular solutions. Recordings were from acutely dissociated MnPO neurons voltage-clamped at −50 mV. B. Summary of the effect of the TRPC channel blockers on the inward current activated by histamine at −50 mV. Cell numbers studied are indicated. ** and * indicate statistical significance of P<0.01 and P<0.05, respectively (ANOVA followed by Tukey-Kramer test) relative to the control.

### Ca^2+^ Imaging

Fura-2 fluorescence signals were acquired with a CCD camera (Hamamatsu ORCA-ER) connected to its frame grabber operating in 8-bit mode and driven by Slidebook software (Intelligent Imaging Innovations, Denver CO USA). An ultra high speed wavelength switcher Lambda DG-4 (Sutter Instruments) equipped with model 340HT15 and 380HT15 filters provided alternating excitation for ratiometric fura-2 measurements. The illumination source was a standard Xenon lamp. The sampling frequency of 0.2 Hz was sufficiently fast given the relatively slow responses to histamine or histamine receptor agonists. At this excitation frequency photobleaching and phototoxicity were minimal. Fura-2AM loading and data acquisition were carried out as described previously [10]. In a group of cells recorded in the whole-cell configuration the pentapotassium salt of fura-2 (Invitrogen, Temecula CA) was loaded through the patch pipette. The final fura-2 concentration in the pipette was 100 µM. The fluorescence signals from ester-loaded cells were in the same range or lower than in cells loaded through the pipette.

**Figure 6:**
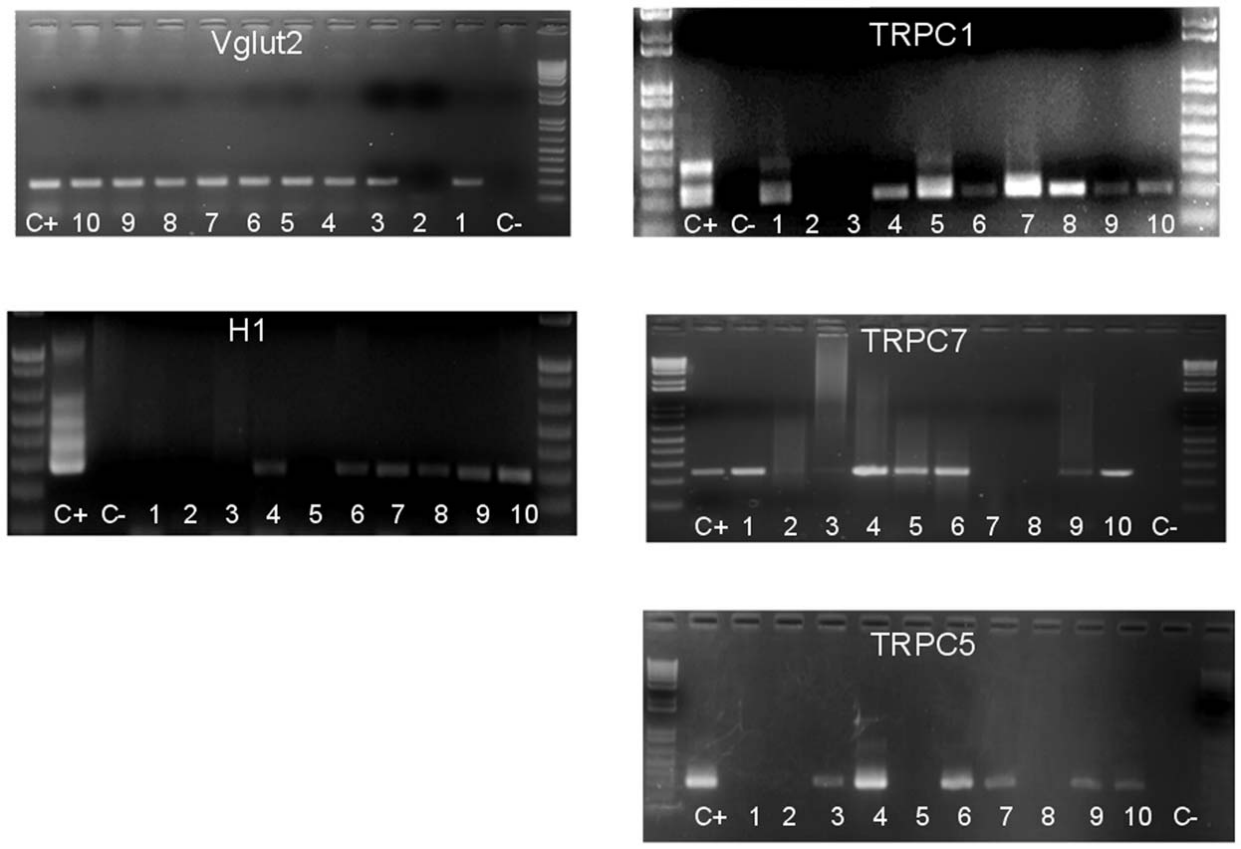
H1 receptor, Vglut2, TRPC1, TRPC7 and TRPC5 transcripts are present in MnPO neurons. Representative gels illustrating the expression of H1 receptor, Vglut2, TRPC1, TRPC7 and TRPC5 transcripts in 10 (1–10) acutely dissociated MnPO neurons. The expected sizes of the PCR products are (in base pairs) 286, 393, 208, 245 and 243, respectively. Negative (−) control was amplified from a harvested cell without reverse-transcription, and positive control (+) was amplified using 1 ng of hypothalamic mRNA. Cells 4 and 6–10 were excited by histamine (50 µM). Cells 1–3 and 5 were not affected by histamine.

### Cell Harvesting and Reverse Transcription

MnPO neurons in slices or dissociated were patch-clamped and then harvested into the patch pipette by applying negative pressure. The content of the pipette was expelled in a PCR tube. dNTPs (0.5 mM), 50 ng random primers (Invitrogen) and H_2_O were added to each cell to a volume of 16 µl. The samples were incubated at 65°C for 5 min and then put on ice for 3 min. First strand buffer (Invitrogen), DTT (5 mM, Invitrogen), RNaseOUT (40 U, Invitrogen) and SuperScriptIII (200 U, Invitrogen) were added to each sample to a volume of 20 µl followed by incubation at room temperature for 5 min, at 50°C for 50 min and then at 75°C for 15 min. After reverse transcription samples were immediately put on ice. 1 µl of RNAse H was added to samples and kept at 37°C for 20 min.

**Figure 7:**
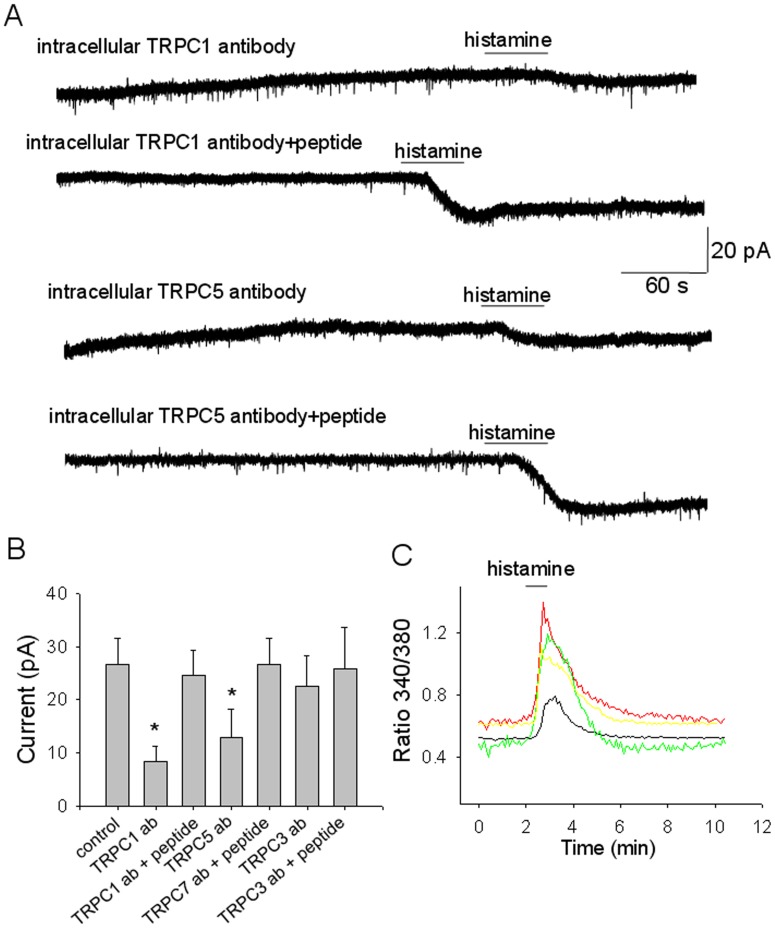
Role of TRPC1 and TRPC5 subunits in the inward current activated by histamine. A. Intracellular application of the TRPC1 antibody (dilution 1∶100) activates an apparent outward current (∼6 pA) and blocks the histamine induced inward current (upper trace). When the antibody was applied intracellularly together with the antigenic peptide the histamine-activated inward current was not affected (lower trace). B. Intracellular application of the TRPC5 antibody (dilution 1∶100) activates an apparent outward current and blocks the histamine induced inward current (upper trace). When the antibody and the antigenic peptide were co-applied intracellularly the histamine-activated inward current was not affected (lower trace). A,B. Recordings were from acutely dissociated MnPO neurons voltage-clamped at -50 mV. TTX (1 µM) was present in the extracellular solution. The downward deflections represent miniature EPSCs from synaptic terminals that remained “attached” to the postsynaptic neuron after dissociation. C. Summary of the effect of the TRPC1, 5 and 3 antibodies applied intracellularly with or without the respective antigenic peptide on the inward current amplitude activated by histamine at −50 mV. The bars represent means ± S.D. (n = 10 for each treatment). * indicates statistical significance of P<0.05 (ANOVA followed by Tukey-Kramer test), when compared to the control. D. [Ca]_i_ responses to histamine (50 µM) from 4 acutely dissociated MnPO neurons pre-incubated for 60 with the TRPC1 antibody. The histamine treatment activated a transient increase in [Ca]_i_ and no plateau.

### Nested PCR

Gene specific primers for H1 and Vglut2 were as described elsewhere [27]. The other primers used are listed in Table 1. cDNAs were amplified in a volume of 25 µl using a high-fidelity Platinum Taq polymerase kit (Invitrogen) and 0.4 mM dNTPs (Invitrogen). For each gene 1/10 of the cDNA from each cell was subjected to a first round of PCR using the outer primer pair and a thermal cycling program with an initial denaturation at 94°C for 2 min, 35 cycles of denaturation at 94°C for 15 s, annealing at 55°C for 30 s and extension at 68°C for 45 s followed by a final extension at 68°C for 10 min. 1.5 µl of the PCR product was subjected to a second round of PCR using the inner primer pair and 40 cycles of the same thermal cycling program as above with the extension time reduced to 30 s. PCR products were visualized by ethidium bromide stained 2% agarose gel electrophoresis.

**Figure 8:**
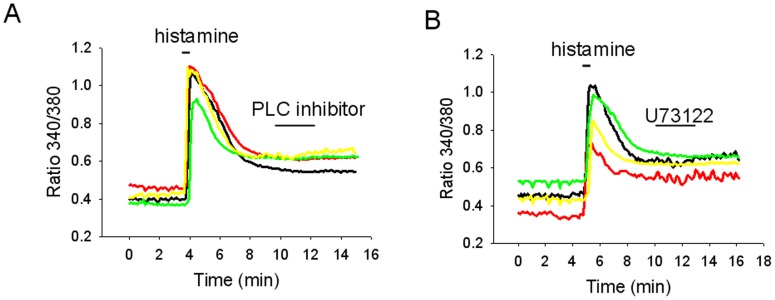
PLCinhibitors do not affect the persistent responses activated by histamine. A. [Ca]_i_ responses to histamine (50 µM) from 4 acutely dissociated MnPO neurons. Incubation with the PLC inhibitor 1 -*O*-octadecyl-2-*O*-methyl-*sn*-glycero-3-phosphorylcholine (10 µM) did not change the “plateau” that followed the histamine-activated transient. B. [Ca]_i_ responses to histamine (50 µM) from 4 acutely dissociated MnPO neurons. The PLC inhibitor U73122 (5 µM) did not affect the persistent response activated by histamine.

## Results

### Transient and Sustained Effects of Histamine in MnPO Glutamatergic Neurons

As reported previously distinct subpopulations of MnPO neurons were excited or inhibited by locally applied histamine [10]. At the onset of this study it was observed that at concentrations higher than 20 µM, the excitatory effect of histamine was only partially reversible even after 20–50 min of wash-out of the agonist (Fig. 1). Thus, a population of MnPO neurons (9 out of 19) recorded in slices was excited by locally applied histamine (50 µM). The spontaneous firing rates averaged 3.2 ± 1.5 Hz (n = 9) and was increased by the neurotransmitter by 254 ± 60% (n = 9) at the peak of the response. The firing rate remained 114 ± 32% (n = 9) higher after 20–25 min wash-out. In voltage-clamp experiments histamine activated an inward current that displayed little recovery even after long periods of washout (20–60 min) (Fig. 2). The current averaged 32 ± 8 pA (n = 14) at the peak and 24 ± 5 pA (n = 14) after 20–25 min washout. In a subgroup of cells, an increase in the frequency of spontaneous (s) EPSCs by 354 ± 170% (n = 4) was also observed. This effect was only partially reversible as well. Thus the sEPSCs frequency remained 94 ± 41% (n = 4) higher after 20–25 min wash-out (Fig. 2A). Re-applying histamine (50 µM) after at least 20 min wash-out resulted again in increased firing rate that then recovered to a level similar to that prior to the second incubation (Fig. 1). In voltage-clamp experiments re-application of the neurotransmitter induced a robust increase in the frequency of sEPSCs and a slight augmentation of the inward current (Fig. 2). The maximal effects tended to be slightly smaller than those obtained during the first application of histamine.

**Figure 9:**
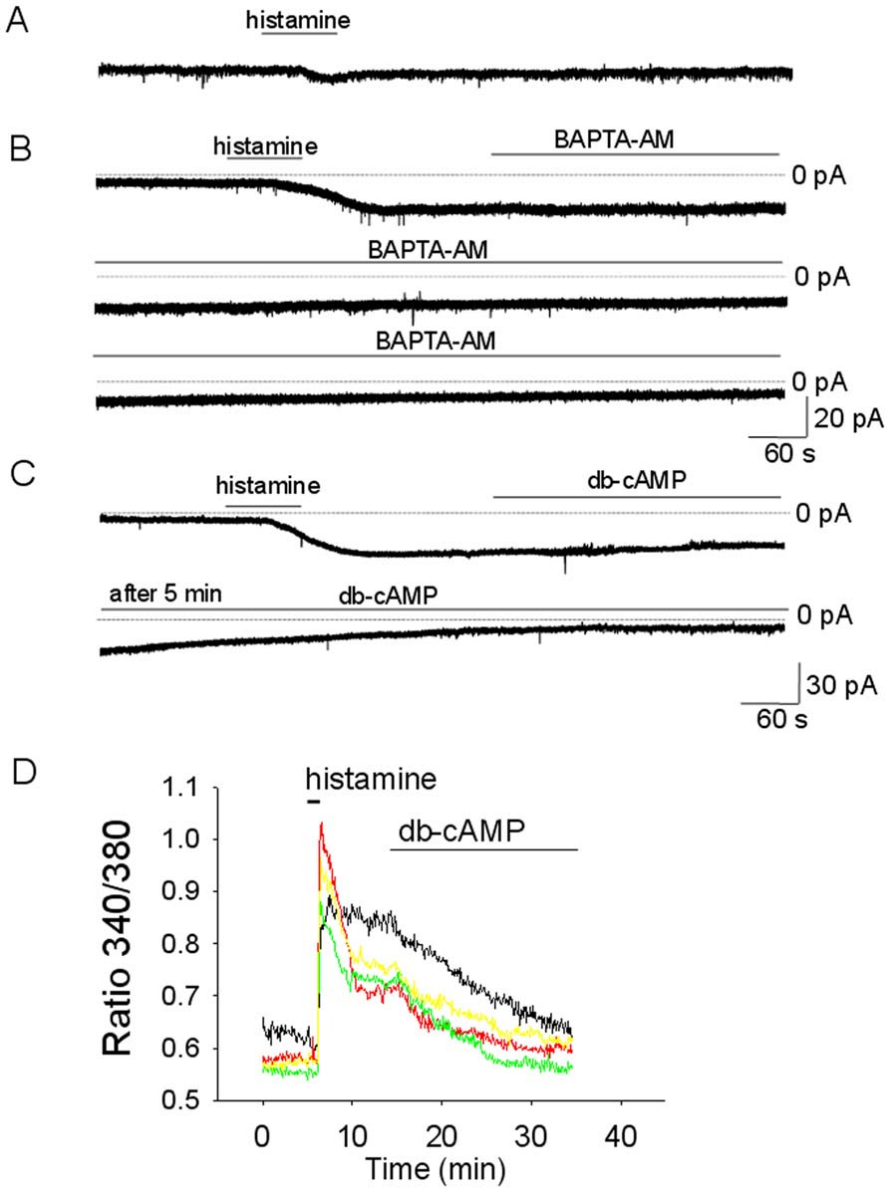
Modulation of the persistent histamine responses by [Ca]_i_ and PKA. A. Histamine response of a MnPO neuron recorded with a pipette solution containing 10 mM BAPTA. B. Extracellular incubation with BAPTA-AM (1 mM) gradually decreases a persistent current activated by histamine (50 µM). C. Incubation with the PKA activator db-cAMP (40 µM) gradually decreases a persistent current activated by histamine (50 µM). D. db-cAMP (40 µM) diminishes the [Ca]_i_ plateau activated by histamine (50 µM). Data from 4 acutely dissociated MnPO neurons. A–C. The neurons were voltage-clamped at −50 mV. A–D. Recordings were from acutely dissociated MnPO neurons. TTX (1 µM) was present in all extracellular solutions.

To ensure that the persistent effects of histamine were not due to incomplete washout of histamine in the slice, similar experiments in acutely dissociated MnPO neurons were carried out. As observed in slices, histamine (50 µM) increased the firing rates of the neurons by 202 ± 31% (n = 13 out of 28 neurons tested) and the firing rate remained elevated by 119±30% (n = 12) after 20 min wash-out. In voltage-clamp measurements the neurotransmitter activated inward currents that displayed little recovery even after 30 min washout in 9 neurons out of 18 tested. The inward current averaged 33 ± 10 pA (n = 9) at the peak and 26 ± 8 pA (n = 9) after 30 min washout. In contrast to the observation in slices, histamine did not affect the frequency of sEPSCs (n = 9), suggesting that the respective effect in slices reflected increased firing activity of a presynaptic neuron. Re-applying histamine (50 µM) after wash-out resulted again in increased firing rate and inward currents that recovered to the plateau reached after the first incubation, observations identical with those in slices.

In contrast to the excitatory effects of the neurotransmitter, the inhibitory action of histamine (50 µM) observed in a distinct set of MnPO dissociated neurons (n = 3 out of 28) were fully reversible within 5–10 min of removal of the agonist. This finding further indicates that the persistent character of histamine excitation was not due to incomplete wash-out of the agonist and raises the possibility that it is a feature of H1 receptor signaling. Histamine (50 µM) did not affect the activity of the remaining cells (n = 12 out of 28).

The I-V characteristics of the histamine-activated inward current were determined by comparing the response to voltage ramps in control conditions and after histamine activated a persistent inward current (Fig. 2B). The difference current reversed at ∼−5 mV and displayed outward rectification. Similar results were obtained in 4 other histamine-excited neurons, the histamine-activated current having a reversal potential of −4.6 ± 1.3 mV (n = 5).

To test whether both the transient and sustained effects of histamine described above involved the activation of H1 receptors we have tested the effect of histamine in cells pre-incubated with the H1 selective antagonists *trans* triprolidine (1 µM) and mepyramine (0.3 µM). In all pre-treated MnPO neurons tested (n = 19 in slices and n = 32 dissociated) the antagonists abolished both the sustained and transient histamine effects (Fig. 3A).

The effect of histamine on the intracellular Ca^2+^ concentrations ([Ca]_i_) was then examined in dissociated MnPO neurons loaded with fura-2AM. Histamine (50 µM) induced a robust increase in [Ca]_i_ in 21% of neurons (32 out of 150). After the first histamine application [Ca]_i_ recovered to a higher level than the control and subsequent histamine incubations elicited transient responses but no further changes in the plateau that followed them (Figs 3B and 4A). This elevated [Ca]_i_ level was constant even over a 80 min washout period. Cells pre-incubated with *trans* triprolidine (1 µM, n = 35) or mepyramine (0.3 µM, n = 26) displayed no change in [Ca]_i_ in response to histamine (Fig. 3B)_._ To determine whether the “plateau” was due to persistent activation of H1 receptors the effect of the two antagonists was tested. The drugs blocked the transient response to histamine but did not change the “plateau” (Fig. 4A) indicating that it reflects the activation of effector proteins downstream of the H1 receptor.

The percentage of MnPO neurons in which [Ca]_i_ was increased by histamine (21%, n = 52 out of 248) was lower than the percentage of neurons excited by the neurotransmitter (47%, see above). This apparent discrepancy is due to the fact that in the electrophysiological recordings we have preselected neurons likely to be nonGABAergic based on their basal firing rates (see Material and Methods) since only nonGABAergic MnPO neurons are excited by histamine [10]. Thus it is expected that the proportion of histamine-responsive neurons is much higher in this subgroup than in the entire population. To directly compare [Ca]_i_ and electrophysiological responses in the same neuron we have carried out simultaneous Ca-imaging and voltage-clamp experiments in a set of dissociated MnPO neurons. In these cells fura-2 (100 µM) was loaded through the patch pipette (see Materials and Methods). We found that 5 out of 20 neurons tested displayed both an increase in [Ca]_i_ and an inward current in response to histamine (50 µM) while the rest (n = 15) did not display any response. Figure 4B (black trace) and Fig. 4C illustrate such an experiment. The inward current averaged 29 ± 6 pA (n = 5). These results confirmed that the inward currents and [Ca]_i_ elevations were activated in the same neurons.

This data establishes that histamine elicits a [Ca]_i_ elevation and an inward cationic current that have both transient and persistent phases. The latter appear to be mediated by effector proteins downstream of the H1 receptor.

### Role of TRPC Channels in the Histamine Actions on MnPO Neurons

TRPC family of channels is commonly associated with signaling pathways of receptors coupled to G_q_, therefore the effects of TRPC channel blocker were tested on the histamine-evoked responses. YM 58483 (100 µM) abolished the plateau and brought the [Ca]_i_ below the control level suggesting that basal TRPC channel activity contributes to [Ca]_i_ (Fig. 4B). The drug did not affect the transient response to histamine (Fig. 4B). Similar results were obtained with another TRPC channel blocker SKF 96365 (100 µM) (Fig. 3C). Either drug had a only a small effect on the basal [Ca]_i._ Thus, YM 58483 (100 µM) and SKF 96365 (100 µM) effects on [Ca]_i_ averaged 17±12% (n = 8) and 15±9% (n = 11), respectively, of the amplitude of their effects in the same cells, when applied during the sustained phase of the histamine response (Fig. 3C).The persistent inward current activated by histamine was potently decreased by YM 58483 (100 µM), SKF 96365 (100 µM) and flufenamic acid (100 µM) (Fig. 4D and Fig. 5 A,B). In contrast, La^3+^ (100 µM) decreased the current by ∼10% (Fig. 5B).

Single cell reverse transcription- PCR (sc RT/PCR) analysis of the TRPC1-7 subunits were then carried out in 41 dissociated MnPO neurons in which histamine effects were tested. Negative (−) control was amplified from a harvested cell without reverse-transcription, and positive control (+) was amplified using 1 ng of hypothalamic mRNA. Other controls (samples of the pipette and bath solutions) were negative after RT-PCR (data not shown). TRPC1 transcripts were detected in all cells in which the neurotransmitter activated an inward current (16 out of 16 neurons). TRPC 5 and TRPC7 were found in 9 and 8 neurons, respectively, out of 16 histamine-excited neurons. In MnPO neurons that were not responsive to histamine (n = 25), TRPC1, TRPC5 and TRPC7 transcripts were found in 13, 11 and 14 neurons, respectively. The TRPC4 subunit was detected in one MnPO neuron excited by histamine and in 4 neurons that were not responsive to the neurotransmitter. The other TRPC subunits were not detected in any neuron tested. Fig. 6 illustrates such an analysis of a group of 10 recorded MnPO neurons. The presence of the Vglut2 and H1 transcripts was also tested. H1 transcripts were found in 14 out of 16 MnPO neurons in which histamine activated an inward current and were not detected in the other neurons studied (n = 27). Vglut2 was detected in all H1 positive neurons and in 33 out of 41 (81%) tested neurons.

To directly address the role of the TRPC subunits in the histamine actions on MnPO neurons blocking antibodies were utilized as described in other studies [21], [23], [24], [25], [26]. Adding TRPC1 antibodies directed against a cytoplasmic site to the pipette solution (dilution 1∶100) resulted in an apparent outward current (6 ± 4 pA, n = 15) that stabilized after 10–15 min of breaking in whole cell- configuration. The inward currents activated by histamine were much smaller than those activated when the antibody was added together with its neutralizing peptide (Fig. 7 A,C). Similar results were obtained with TRPC5 antibodies directed against a cytoplasmic site (Fig. 7 B, C), albeit the effect appeared to be smaller than that of the TRPC1 antibody (P>0.05 ANOVA). As a negative control the effect of a TRPC3 antibody directed against a cytoplasmic site was determined, since this subunit was not detected in MnPO neurons. This antibody (with or without blocking peptide) had no effect on the amplitude of the histamine-activated inward current (Fig. 8C). Furthermore, the TRPC3 specific blocker Pyr3 (20 µM) was without effect on the inward current or the [Ca]_i_ plateau activated by histamine (n = 8 and n = 12, respectively), confirming that this subunit was not involved in the histamine responses of MnPO neurons.

The effect of the TRPC1 antibody when applied in the extracellular solution (dilution 1∶100) was also measured as described by others [25]. Incubated cells (for 60 min) displayed only transient responses to histamine (50 µM, n = 11) (Fig. 6D) and no inward currents (n = 15, data not shown).

These results indicate that TRPC channels, in particular TRPC1 and 5 and not TRPC3, are activated during the “plateau” phase of the histamine response.

### Signaling Pathways Regulating the Sustained Effects of Histamine in MnPO Neurons

H1 receptor signaling is usually associated with the PLC pathway (reviewed in [28]). In a previous study we have shown that both the elevation of [Ca]_i_ and the inward current were blocked by pre-incubation with PLC inhibitors [10]. However, neither U73122 (5 µM, n = 7) nor 1-O-Octadecyl-2-O-methyl-*sn*-glycero-3-phosphorylcholine (10 µM, n = 5) changed the sustained inward current once it was activated (Fig. 8). Similarly, the inhibitors did not change the [Ca]_i_ plateau (n = 10 and n = 5, respectively). The role of pathways that modulate TRPC channel activity, such as [Ca]_i_ and PKA, in the sustained effects of histamine was then studied.

Recordings with a pipette solution containing 10 mM BAPTA were characterized by small inward currents evoked by histamine (50 µM), 8±3 pA (n = 9, P<0.01 unpaired t- test, compared to recordings with the control pipette solution) with no persistent component (Fig. 9A), indicating that increased [Ca]_i_ was necessary for this effect. In a set of experiments BAPTA-AM (1 mM) was applied in the extracellular solution after a persistent current was activated by histamine. The incubation resulted in a gradual decrease in the persistent inward current (Fig. 9B). The amplitude of the current was significantly reduced (P<0.05, paired t-test) after 30 min of BAPTA-AM incubation, averaging 27±16% of the control value (n = 5).

PKA activation with db-cAMP (40 µM) resulted in a gradual decrease in both the sustained inward current as well as the [Ca]_i_ plateau (Fig. 9C,D). After 20 min of db-cAMP (40 µM) incubation the persistent current was decreased by 75±16% (n = 6). Similarly the [Ca]_i_ plateau was reduced by 79±18% (n = 7). Conversely, preincubation with the PKA inhibitor KT5720 (1 µM) resulted in increased persistent currents evoked by histamine (50 µM), 42±7 pA (n = 8, P<0.05) and increased [Ca]_i_ plateaus. The [Ca]_i_ plateau averaged 40±13% (n = 15) of the peak response in control experiments and 69±16% (n = 11) (P<0.05, unpaired t-test) in KT5720 pre-treated neurons.

This data establishes that elevation of [Ca]i is required for the activation of a persistent inward current by histamine. It also shows that a PKA pathway negatively regulates the persistent phases of both the [Ca]_i_ and inward current responses.

## Discussion

In this study we reveal a novel mechanism by which histamine activates sustained excitation of H1R-expressing glutamatergic MnPO neurons. The neurotransmitter activated inward currents and [Ca]_i_ elevations that have both transient and “plateau” phases. The latter (unlike the inhibitory effects of histamine in a GABAergic population of MnPO neurons [6]) do not recover even after 20–60 min of washout. The data reported here also establishes the role of the TRPC family of channels (in particular TRPC1 and TRPC5) in the inward current and [Ca]_i_ elevation activated by histamine in MnPO neurons. We also reveal that the persistent phases of the responses are negatively regulated by PKA. Interestingly, a sustained effect of histamine acting at H1 receptors on the secretion of gonadotropin-releasing hormone, with similar time course, has been reported in immortalized hypothalamic neurons [29].

Persistent activity, a characteristic of a neuron in which a brief stimulus induces a long lasting response, is generally associated with recurrent excitatory networks [30] or with autaptic synapses [31]. The present report identifies a novel mechanism for inducing persistent activity that is “postsynaptic”. In slices the excitation was accompanied by an increase in sEPSC frequency in a subpopulation of MnPO neurons, an effect probably due to reciprocal connections of MnPO glutamatergic neurons [10]. In isolated neurons this effect was never observed ruling out the involvement of autapses. The increase in sEPSC frequency had a sustained component as well, likely a consequence of elevated [Ca]_i_ in the presynaptic neuron.

Several characteristics of the persistent inward current activated by histamine indicate a role of the TRPC family of channels. The outward rectification and reversal potential were similar to those reported for TRPC1-containing heteromers expressed in HEK293 cells [32], [33]. The sensitivity of the histamine-activated inward currents to YM 58483, SKF 96365 and flufenamic acid clearly suggests an involvement of this class of subunits. These compounds have unrelated structures and, although their specificity for TRPC channels appears unlikely, they share their blocking action at these channels. YM 58483 is blocker with marked selectivity for store-operated Ca^2+^ entry [34], [35], [36], [37], [38]. Its inhibitory action at TRPC1, 3 and 6 channels has been documented directly [35], [37], [39]. While the role of TRPC1-7 subunits in store-operated Ca^2+^ entry in various cell types has been documented, direct evidence of YM 58483 inhibition of the other TRPC subunits is not available. YM 58483 does not block voltage-dependent Ca^2+^ channels or TRPV channels [35], however its specificity for TRPC-containing channels remains to be established. Similarly, SKF 96365 was identified as a blocker of store-operated Ca^2+^ entry [40], [41] and nonselective cationic currents in various cell types [42], [43]. Evidence for blocking actions at TRPC1 [44], TRPC3 [45], TRPC5 [46], TRPC6 [47], [48] and TRPC7 [45] channels has accumulated. SKF 96365 was reported to block also voltage-gated Ca^2+^ channels [41]. Flufenamic acid is a clinically used nonsteroidal anti-inflammatory drug and also modulates multiple ion channel activities, including nonselective cation channels [49] such as TRPC channels [50] and TRPM channels [51]. Flufenamic acid blocks TRPC3 and TRPC7 channels and potentiates TRPC6-channels [50]. Its action at the other TRPC subunits has not been determined directly so far.

We have used intracellular application of antibodies directed against cytoplasmic domains of the TRPC 1, 3 and 5 subunits to establish their contributions to the histamine-induced inward currents. This procedure has been used in numerous studies [21], [22], [23], [24], [25], [26]. It is believed that the binding of the antibody prevents conformational changes related to channel gating. The specificity of these antibodies for the respective epitopes is well documented based on Western blots and peptide absorption experiments. The intracellular concentration of the antibodies used in this study is estimated to be ∼ 8 µg/mL (1∶100 dilution) because the patch pipette solution and the cytoplasm equilibrate within 10–15 min of establishing the whole-cell configuration. Because of the high concentrations of antibodies used the possibility of cross reactivity with other proteins that contain a similar epitope cannot be ruled out. However in situation in which antibodies directed against different epitopes have been tested the blocking action was similar [22].

In this study, TRPC1 transcripts were detected in all histamine-responsive neurons as well as TRPC5 and/or TRPC7 in a majority of them. It appears that MnPO neurons express a heterogenous complement of TRPC subunits, as found also for other neuronal types [42], [52]. Our experiments using blocking antibodies confirmed the central role played by the TRPC1 subunit in the inward current activated by histamine and smaller contributions of TRPC5 subunits. While the persistent component of the [Ca]_i_ response was abolished by block of TRPC1 the transient one was little affected, indicating that Ca influx through TRPC1 contributes little to it. Since TRPC1 subunits cannot form functional channels, at least not in HEK293 cells [33], it appears likely that the inward current activated by histamine in MnPO neurons are conducted by TRPC1/5 and/or TRPC1/7 heteromers. The fact that La^3+^ had little effect on these currents would suggest that both types of channels are present, because the cation is expected to have opposing effects on them: potentiation of TRPC5-containing channels [53] and block of TRPC7-containing channels [54].

Activation of an inward current by histamine requires the activation of the PLC pathway [10]. This study shows that once the current was activated the PLC activity was no longer neccesary. An increased [Ca]_i_ was required for both the activation of the persistent current, as reported for TRPC1/5 channels [55], as well as for its maintenance. This report also reveals that activation of the PKA pathway has an inhibitory effect on the histamine-activated current. Conversely, inhibiting PKA activity resulted in increased inward current and [Ca]_i_ plateau. These effects were probably due to direct action on the activity of the TRPC channels that are inhibited by PKA phosphorylation [56], [57]. These observations suggest that *in vivo* such persistent activity could be reset by signaling mechanisms that result in PKA activation or in a reduction of [Ca]_i_.

In summary, this study elucidates a cellular mechanism by which histamine induces long lasting excitation of glutamatergic MnPO neurons that appears to involve elevation of [Ca]_i_ and activation of TRPC channels.
